# Elucidating the neuroimmunology of traumatic brain injury: methodological approaches to unravel intercellular communication and function

**DOI:** 10.3389/fncel.2023.1322325

**Published:** 2023-12-13

**Authors:** Hadi Abou-El-Hassan, Joshua D. Bernstock, Joshua I. Chalif, Taha Yahya, Rafael M. Rezende, Howard L. Weiner, Saef Izzy

**Affiliations:** ^1^Ann Romney Center for Neurologic Diseases, Brigham and Women’s Hospital, Harvard Medical School, Boston, MA, United States; ^2^Department of Neurosurgery, Brigham and Women’s Hospital, Harvard Medical School, Boston, MA, United States; ^3^David H. Koch Institute for Integrative Cancer Research, Massachusetts Institute of Technology, Cambridge, MA, United States

**Keywords:** traumatic brain injury, neuroimmunology, neurodegeneration, neuroinflammation, lymphocytes, neuroglia

## Abstract

The neuroimmunology of traumatic brain injury (TBI) has recently gained recognition as a crucial element in the secondary pathophysiological consequences that occur following neurotrauma. Both immune cells residing within the central nervous system (CNS) and those migrating from the periphery play significant roles in the development of secondary brain injury. However, the precise mechanisms governing communication between innate and adaptive immune cells remain incompletely understood, partly due to a limited utilization of relevant experimental models and techniques. Therefore, in this discussion, we outline current methodologies that can aid in the exploration of TBI neuroimmunology, with a particular emphasis on the interactions between resident neuroglial cells and recruited lymphocytes. These techniques encompass adoptive cell transfer, intra-CNS injection(s), selective cellular depletion, genetic manipulation, molecular neuroimaging, as well as *in vitro* co-culture systems and the utilization of organoid models. By incorporating key elements of both innate and adaptive immunity, these methods facilitate the examination of clinically relevant interactions. In addition to these preclinical approaches, we also detail an emerging avenue of research that seeks to leverage human biofluids. This approach enables the investigation of how resident and infiltrating immune cells modulate neuroglial responses after TBI. Considering the growing significance of neuroinflammation in TBI, the introduction and application of advanced methodologies will be pivotal in advancing translational research in this field.

## Introduction

Traumatic brain injury (TBI) continues to be a leading cause of morbidity and mortality (Maas et al., 2008). Globally, it is estimated that 69 million individuals are diagnosed with TBI annually (Hammond F. M. et al., 2019), among which approximately 1.7 million occur in the United States (Georges and Das, 2023). The socioeconomic burden of TBI is profound given its association with mortality and debilitating chronic neurological, psychological and cardiovascular (Izzy et al., 2022). Neurodegeneration plays an important role in the pathogenesis of TBI (McKee et al., 2015) and is governed by an array of molecular and cellular mechanisms that ultimately contribute to the development of neurocognitive deficits among TBI survivors (Abou-El-Hassan et al., 2017a).

It is prudent to note that TBI is heterogeneous in nature and the underlying pathobiology is inherently complex (Jassam et al., 2017). TBI causes a primary injury followed by a secondary biochemical and cellular response, which involves the induction of the neuroinflammatory response (Jassam et al., 2017). The neuroglia present within the central nervous system (CNS), that play a pivotal role in maintaining neuroglial homeostasis and neurovascular integrity (Nasser et al., 2016; Abou-El-Hassan et al., 2017b,^2020^; Nokkari et al., 2018), can be activated and altered in the setting of traumatic injuries. The infiltration of immune cells from the periphery (e.g., T cells) and their ultimate pathologic crosstalk with neuroglia further facilitates TBI pathology and neurodegeneration (Jassam et al., 2017; Abou-El-Hassan et al., 2023). The involvement of microglia, infiltrated monocytes and leucocytes have been observed in animal and human TBI studies. However, the investigations in TBI have been hampered by the lack of the understanding of the exact mechanisms governing innate-adaptive immune response crosstalk (i.e., between lymphocytes and neuroglia, such as microglia and astrocytes) and limited utilization of relevant experimental models/methods.

Traumatic brain injury (TBI) survivors suffer from long-term disabling impairments in cognition, sensorimotor function, and behavioral changes (Lipton et al., 2008; Galea et al., 2022). Several animal models of TBI, as detailed elsewhere (Xiong et al., 2013), have been developed over the past decades to mimic the various types and severities of human brain injuries. TBI models are either open head injury by subjecting the animal to a physical injury directly to the actual brain parenchyma such as the controlled cortical impact (CCI) model, or a closed head injury model whereby the brain tissue is spared from any direct injury such as the weight drop model. Altogether, such models have been developed to recapitulate the various aspects of acute and chronic characteristics of TBI at the cellular and phenotypic levels with the goal of better understanding the underlying TBI pathophysiology and help guide the development of neurotherapeutics. Nevertheless, experimental drugs that were found to be neuroprotective at the preclinical stage, have failed in the advanced phases of TBI clinical trials. This failure at the translational level provides compelling evidence on the need to revisit the current status of models and methods of pertaining to TBI investigations.

**FIGURE 1:**
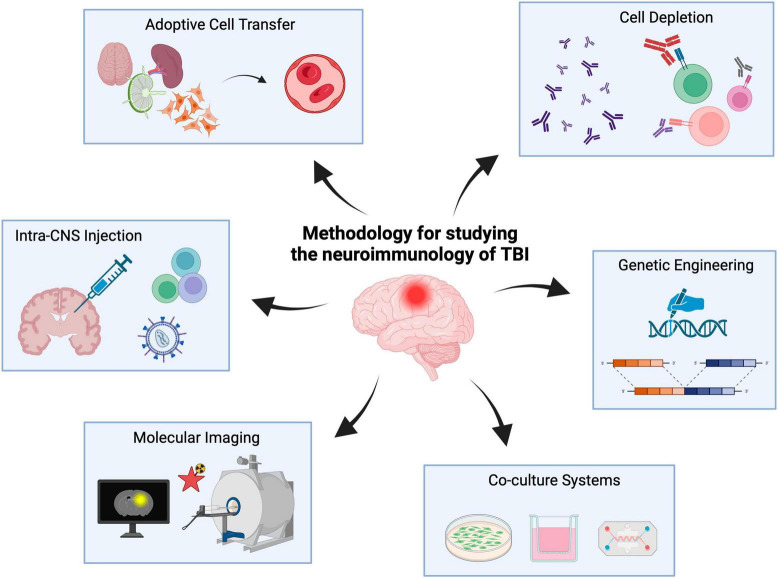
Experimental methods to study the neuroimmunology of TBI. Neuroimmunology methods include adoptive cell transfer, cell depletion, intra-CNS injections, genetic engineering, co-culture systems and molecular imaging. Created with biorender.com.

Accordingly, herein we describe methods and approaches capable of facilitating the study of the neuroimmunology underlying TBI with a focus on neuroglia-lymphocyte crosstalk. These methods include adoptive cell transfer, intra-CNS injections, cell depletion, tissue- and temporal-specific genetic manipulations, molecular neuroimaging as well as *in vitro* co-culture systems (Figure 1). It is important to note that this body of work is not meant to serve as a systematic review of the neuroimmunology of TBI as has been described elsewhere (Jassam et al., 2017; Bouras et al., 2022) but rather a methodological overview of the approaches currently used to study TBI neuroimmunology with an eye toward the future (i.e., both preclinical and clinical).

## Adoptive cell transfer

Adoptive cell transfer (ACT) is one of the commonly employed experimental techniques in immunology. This method involves isolating a specific cell population and subsequently introducing it into a recipient host. In animal models of disease, this transfer is typically accomplished through intravenous or intraperitoneal routes (direct intra-CNS injection, referred to as “CNS ACT,” is discussed separately below) (Rosenberg et al., 2008). Before the transfer, the isolated cell population may undergo culture in suitable media for a certain period. However, with today’s cell sorting technology, it’s possible to immediately isolate and transfer live cells, bypassing the need for *ex vivo* culturing steps (Baron et al., 2019). In cases where a substantial number of cells is required for ACT, expansion through cell culture is often essential.

Historically, ACT-based immunotherapy was first described in 1988 for the treatment of patients with metastatic melanoma (Dudley et al., 2005). For example, clonal repopulation of tumor-infiltrating lymphocytes have been used in such oncological applications (Dudley et al., 2002) along with the use of autologous regulatory T cells (Tregs) in autoimmune diseases (Bluestone et al., 2015; Canavan et al., 2016). Since then, several modifications have been suggested/employed whereby donor cells are modified in an effort to amplify their desired therapeutic role(s). More recently, lymphocytes were genetically modified to identify immune antigens to facilitate the disease regression (Morgan et al., 2006). For example, tumor regression was observed in mice receiving engineered splenocytes expressing an anti-melanoma T-cell receptor (TCR) (Abad et al., 2008) and prevention of allograft rejection was achieved using chimeric antigen receptor (CAR) Treg cell therapy in experimental allogeneic transplantation (Noyan et al., 2017).

Given the correlation between T cell infiltration and neurodegeneration (Daglas et al., 2019) in addition to our work centered on defining the role(s) of lymphocyte subsets in TBI (Abou-El-Hassan et al., 2023), it is imperative to further highlight methods capable of integrating/exploring immunological mechanisms driving the sequela of brain injury. In the setting of TBI, very few studies have attempted ACT of T cells or T cell subsets (Table 1). These include three main studies, described below, investigating the effect of adoptively transferring CD4^+^CD62L*^low^*CD44*^high^* T cells (Fee et al., 2003), bone marrow-derived monocytes (Braun et al., 2017), and γδ T cell subsets (Abou-El-Hassan et al., 2023) after TBI.

**TABLE 1:** A sample of TBI neuroimmunology studies categorized by methodological approach.

Intervention	Method	Timepoint	Number of cells/dose	Model of TBI	Recipient species	Outcomes	References
**Adoptive transfer**							
CD4^+^CD62L*^low^*CD44^high^ T cells from spleen	ICV injection	24 h prior to injury	2 × 10^6^ cells per mouse	aseptic cerebral injury	RAG1^–/–^ mice	Worsened cerebral injury at 24 h post-injury	Fee et al., 2003
CD11b^+^ CD68^+^ F4/80^+^ macrophages from bone marrow and spleen	ICV injection	Onset of TBI	2 × 10^5^ cells per mouse	CCI	C57BL/6J mice	Increased polarization of M1 macrophages at 72 h post-injury	Braun et al., 2017
Vγ1 γδ T cells from spleen	IV injection	Onset of TBI	5 × 10^5^ cells per mouse	CCI	TCRδ^–/–^ mice	Modulated neuroinflammation at 48 h post-injury	Abou-El-Hassan et al., 2023
Vγ4 γδ T cells from spleen	IV injection	Onset of TBI	5 × 10^5^ cells per mouse	CCI	TCRδ^–/–^ mice	Worsened neuroinflammation at 48 h post-injury	Abou-El-Hassan et al., 2023
**Cell depletion**							
Anti-CD8 depletion antibody	IP injection	(day 1–3), day 8 and 15. 4 weeks after TBI	0.1 mg in 200 μL	CCI	C57BL/6J mice	Improved neurological recovery at between 4 and 8 weeks post-injury.	Daglas et al., 2019
Macrophage/microglia depletion using clodronate liposomes	IP injection	3 straight days prior to TBI	5 mg/mL in 200 μL	CCI	C57BL/6J mice	Reduced Th1/Th17 polarization at 72 h post-injury in brain and blood	Braun et al., 2017
Anti-Vγ1 depletion antibody	IP injection	24 h before TBI	200 μg in 200 μL	CCI	TCRδ^–/–^ mice	Modulated neuroinflammation at 48 h post-injury	Abou-El-Hassan et al., 2023
Anti-Vγ4 depletion antibody	IP injection	24 h before TBI	200 μg in 200 μL	CCI	TCRδ^–/–^ mice	Reduced neuroinflammation at 48 h post-injury	Abou-El-Hassan et al., 2023
Diphtheria toxin-induced depletion of Tregs	IP injection	24 h before and 24 h after TBI	1 μg	CCI	C57BL/6J DEREG- mice	Worsened neuroinflammation and neurological score between 1 and 5 days post-injury	Krämer et al., 2019
Anti-CD25 depletion antibody	IP injection	48 h prior to TBI	200 μg	CCI	C57BL/6J	Enlarged lesion volume and exacerbated sensorimotor deficits at 5 days post-injury	Xie et al., 2022
Anti-Gr-1 depletion antibody	IP injection	12 h prior and 12 h after TBI	100 μg	CCI	C57BL/6J mice	Reduced edema at 24 h/48 h. Reduced microglia/macrophage activation at 7 days and reduced lesion volume at 7- and 14-days post TBI.	Kenne et al., 2012
Valganciclovir-induced CD11b^+^ depletion	ICV osmotic pump	7 days before and 7 or 21 days after TBI	(low)1 mg/mL (Inter)10 mg/mL (High)50 mg/mL	rCHI	CD11b-TK (thymidine kinase) mice	No effect on silver staining, amyloid precursor protein accumulation, neurofilament labeling, or axonal injury	Bennett and Brody, 2014
Diphtheria toxin-induced depletion of CD11b^+^ cells	IP injection	24 h before and 24 h after TBI	20 ng/g	CCI	CD11b-DTR mice	Increase in proinflammatory gene expression in the brain at 48/72 h after injury.	Frieler et al., 2015
Macrophage/microglia depletion using clodronate liposomes	IC injection	24 h after TBI	10 μL of 5 mg/ml	CCI	Sprague-Dawley rat pups	Increased neurodegeneration at 3 days and fluoro-Jade B reactivity at 2–4 weeks post-injury.	Hanlon et al., 2019
Macrophage/monocyte depletion using clodronate liposomes	IV injection	18 h before or 18 h before/after and 3 days after TBI	10 μL/g Body weight	CCI	C57BL/6J mice	Abrogated the neuroprotective effects of B-cell adoptive cell transfer	Dwyer et al., 2023
**Direct intra-CNS injection**							
B cell injection	Intraparenchymal at lesion site	Immediately before TBI	2.5 × 10^6^ cells per mouse	CCI	C57BL/6J mice	Reduced lesion volume, microglia and astrocyte activation, and motor and memory up to 35 days post TBI	Sîrbulescu et al., 2019
B cell injection	Intraparenchymal at lesion site	Immediately before TBI	2.5 × 10^6^ cells per mouse	CCI	C57BL/6J mice	Significantly increased expression of IL-10, IL-35, and TGFβ on myeloid cells and reduced microglia activation. From 4 days and upto 2 months post-injury	Dwyer et al., 2023
Aquaporin-4 shRNA	Bilateral intraventricular injection	30 min, 12 h, 24 h and every other day post-TBI	10 uL of 15 ug of virus dissolved in phosphate-buffered saline	Hydraulic craniocerebral trauma	Rats	Reduced brain edema, neuronal apoptosis, astrocyte activation at 3 days post TBI and neurological deficits up to 14 days post TBI	Li et al., 2022
β-Ngf pseudo lentivirus	Bilateral intraventricular injection	Onset of TBI	10 uL of 1 × 10^7^ TU/mL	CCI	Rats	Increased neurite growth starting at 3 days and up to 28 days post-injection and improved memory at 14 days post-injection	Lin et al., 2015
IFN-γR1 lentivirus targeting CD11b^+^ cells	Bilateral intraventricular injection	–	1 × 10^7^ IU	CCI	C57BL/6J mice	Increase in microglia homeostatic profile at 2 days post-injury	Abou-El-Hassan et al., 2023
**Genetic engineering**							
Inducible global HMGB1 knockout	IP injection of tamoxifen	6–7 weeks of tamoxifen in mice aged 6 weeks or older	–	CCI	HMGB1^fl/fl^ mice	Reduction in lesion volume at 21 days post-injury	Aneja et al., 2019
Inducible deletion of *Nhe1* in Cx3cr1+ cells	IP injection of tamoxifen	Postnatal day (30–40) for 5 straight days and then a 30 day wait period for replenishment of Cx3cr1+ monocytes	75 mg/kg body weight/day at a concentration of 20 mg/m	CCI	*Cx3cr1CreER^±^;Nhe1^f/f^* mice	Accelerated oligodendrocyte regeneration. Increased anti and reduced pro-inflammatory microglia/infiltrated myeloid cells transcriptomic profiles. Improved sensorimotor and cognitive	Song et al., 2022
**Co-culture systems**							
3D co-culture of cortical neurons using a silk scaffold embedded in collagen	Mouse cortical neurons were grown on a silk scaffold embedded in collagen and were cultured for 14 days	3D co-culture was injured at 14 days after culture	–	CCI	–	Degradation of neural network structure and necrosis marker upregulation was observed during the 24 h window after injury	Liaudanskaya et al., 2020
Culture of stem cell-derived microglia	Addition of IL-1β, IL-4, IL-6, IL-10, TNF	–	–	–	–	Downstream cytokine response was measured over a 72 h time course period during exposure	Alam et al., 2023
Direct co-culture of primary microglia and oligodendrocytes	LPS or LPS plus IFN-γ was added to microglia for M1 induction. IL-4 was added for M2 induction.	–	–	Scriptaid (histone deacetylases inhibitor)	–	M2 microglia preserved oligodendrocytes viability after 24 h of transwell co-culture	Wang et al., 2015
Transwell co-culture of microglia and Vγ1 γδ T cells + anti-TGF-	A transwell system: Vγ1 γδ T cells (top chamber) and microglia (bottom chamber)	Sorted microglia 24 h post-injury and Vγ1 γδ T cells from spleens of naïve mice	1 × 10^5^ microglia cells and 5 × 10^4^ Vγ1 γδ T cells. 10 μg/mL of anti-TGF-β	CCI	–	Activation of microglia pro-inflammatory transcriptomic signature after 48 h of co-culture	Abou-El-Hassan et al., 2023
Transwell co-culture of microglia and Vγ4 γδ T cells + anti-IL-17A	A transwell system: Vγ4 γδ T cells (top chamber) and microglia (bottom chamber)	Sorted microglia 24 h post-injury and Vγ4 γδ T cells from spleens of naïve mice	1 × 10^5^ microglia cells and 5 × 10^4^ Vγ1 γδ T cells. 10 μg/mL of anti-IL-17A	CCI	–	Attenuation of microglia pro-inflammatory transcriptomic signature after 48 h of co-culture	Abou-El-Hassan et al., 2023

Knowing that the adaptive immune system is activated in TBI (Jassam et al., 2017), the adoptive transfer of effector CD4^+^CD62L*^low^*CD44*^high^* T cells 24 h prior to injury into RAG1^–/–^ mice (which do not contain mature B or T cells), resulted in significant worsening of the cerebral injury at 24 h after TBI as compared to control C57BL/6 control mice (Fee et al., 2003); in contrast, transfer of CD4^+^CD62L*^high^*CD44*^low^* T cells did not result in the degree of injury. This is consistent with the fact that CD44 has been used as a prominent activation marker that distinguishes effector T cells from their naïve counterparts (Schumann et al., 2015). Of note, the transferred cells were also found to localize around the lesion site post-injury confirming their direct effect(s) within the perilesional brain tissue. While these results did not detail the interaction between neuroglia and CD4^+^CD62L*^low^*CD44*^high^* T cells, these findings suggest the involvement of the adaptive immune system in acute TBI, where specific CD4 T cell subsets are capable of driving pathogenic inflammation (Guan et al., 2011).

Beyond driving injury, the subset of T cells that have been shown capable of ameliorating neuroinflammation after TBI is Tregs. Tregs are known to play an important role in buffering/modulating inflammation (Vignali et al., 2008). In animal models of stroke (Li et al., 2013), post-stroke hemorrhage (Mao L. et al., 2017) and intracerebral hemorrhage (Mao L.-L. et al., 2017), the adoptive transfer of Tregs has reduced neuroinflammation and improved preclinical outcomes. Unfortunately, such investigation(s) have not been extended to experimental TBI but given the underlying biology it would be reasonable to expect similar outcome(s). The increased number of activated macrophages following the adoptive transfer of monocytes warrants potentiating the regulatory arm of the adaptive immune system to alleviate the TBI neuropathology (Braun et al., 2017). Further, given that in stroke patients, the number of circulating Tregs decreases dramatically post-stroke (Urra et al., 2009), investigating whether Tregs and the regulatory factors produced, such as IL-10 or TGF-β, ameliorate resultant neuroinflammation/neurodegeneration after TBI is clearly warranted (Jassam et al., 2017).

Recent work has also shed light on gamma-delta (γδ) T cells as major drivers of neuroinflammation (Shichita et al., 2009; Sun et al., 2018). γδ T cells are more abundant in non-CNS compartments and their frequency is lower than that of αβ T cells within the CNS (Rezende et al., 2018). Our lab has recently reported on the opposing roles of γδ T cell subsets, Vγ1 and Vγ4, after TBI (Abou-El-Hassan et al., 2023). We found that Vγ1 γδ T cells are protective due to the secretion of TGF-β whereas Vγ4 γδ T cells potentiate pathogenic inflammation following TBI via the secretion IFN-γ and IL-17. In fact, the adoptive transfer of Vγ1 γδ T cells into injured TCRδ^–/–^ mice ameliorated neuroinflammation whereas the adoptive transfer of Vγ4 γδ T cells worsened neuroinflammation (Abou-El-Hassan et al., 2023). We found that Vγ1 γδ T cells crosstalk with microglia resulting in a decrease in microglial stress genes along with an upregulation of homeostatic genes. In line with such findings, the adoptive transfer of Vγ1 γδ T cells into injured wild-type mice improved neurological function after TBI.

The referenced studies that employed ACT provided evidence related to the involvement of the adaptive immunity system in TBI. The temporal role of the adaptive immune system throughout the natural course of TBI is not fully understood and time-specific ACT-based immunoassays would help to further investigate the roles of the adaptive immune cells. Further immunoassays and applications would include transwell co-culture assays, cell death assays and phagocytosis assays. In acute TBI thus far, isolating Tregs or Vγ1 γδ T cells from patients hospitalized with acute TBI and re-administering such cells into those same patients using a CAR T-cell therapy-like approach, may therefore represent a promising immunotherapy-centered approach for TBI. Further preclinical and ultimately clinical research is warranted to investigate lymphocyte-neuroglia crosstalk mechanisms using ACT assays in cell- and time-specific fashion.

Among the advantages of ACT is that it allows for highly targeted investigations of a particular cell population of interest, with the ability to conduct precise cellular expansions and modifications. Complexity, time-consuming and regulatory challenges are among the challenges of ACT applications.

## Direct intra-CNS injections

The direct intra-CNS injection of chosen immune cells, referred here to as CNS ACT, along with immunomodulators, for studying TBI neuroimmunology and as an immunotherapy, has not been widely employed. This could be attributed in part to the technical expertise and invasive nature required for intra-CNS injections. One primary advantage of direct CNS injections is their ability to bypass the blood-brain barrier (BBB), simplifying the delivery of cells or pharmacological agents. In preclinical experiments, these injections typically involve carefully passing a syringe through soft tissue to administer a specific cell population into the brain parenchyma or cerebrospinal fluid. In a clinical context, CNS injections are feasible, especially in patients with external ventricular drains and similar ventriculostomies. It’s crucial to note that determining the appropriate quantity of cells to inject or the dosage of a particular modulator often necessitates an in-house optimization process.

Few preclinical TBI studies have employed the direct intra-CNS injection approach to assess the role of B and T lymphocytes and their role in outcomes (Table 1). These studies, as described below, have been limited to the injection of B cells and that of various immunomodulating lentiviruses (discussed in the genetic engineering section).

Regarding B cells, intraparenchymal administration of B cells resulted in improved motor coordination and memory in C57BL/6 mice using the CCI model of TBI (Sîrbulescu et al., 2019), suggesting that B cells may play a neuroprotective role in acute brain injury. This is in contrast to cytotoxic CD8^+^ T cells that were found to promote neurodegeneration after TBI (Daglas et al., 2019). Moreover, lesion volume in B cell-recipients was significantly decreased as compared to controls. In a separate investigation, B cell subsets expressing IL-10 or TGF-β dominated at 10 days *in situ* (Dwyer et al., 2023). In fact, proteomic analysis revealed that B cells likely exhibit a homeostatic function in the injured microenvironment as evident by the overexpression of genes involved in tissue remodeling and oxidative protection (Sîrbulescu et al., 2021). Furthermore, fewer TNF-α, IFN-γ and IL-6 producing cells were detected in mice treated with 1 × 10^7^ B cells as well as increased number of CD206^+^ infiltrating monocytes/macrophages (Dwyer et al., 2023). Interestingly, depletion of peripheral monocytes abrogated the regulatory phenotype of exogenous B cells suggesting that crosstalk between the activated adaptive and innate immune systems is required for the neuroprotective immunomodulatory effects of exogenous B cells.

Delineating the definite crosstalk mechanisms between the adaptive and innate immune responses requires advanced cell-specific technology such as single-cell RNA-seq. Using intra-CNS injections and other neuroimmunology assays will enable such investigations. The dynamic nature of TBI neuroimmunology across the various phases of TBI (acute, subacute, and chronic) requires temporal analysis of those molecular and cellular changes. Although intra-CNS injections prior to TBI may be an invasive approach from a translational standpoint in mild or moderate TBIs, they provide immunological insights and may confirm the inter-cellular mechanisms involved. Nevertheless, the use of intra-CNS injections after TBI should not be limited by invasiveness given the feasibility of these approaches and the therapeutic potential of, for example, regulatory cells of the adaptive immune system. For example, intra-CNS injection of Tregs has never been investigated and may theoretically offer a faster and more effective recovery compared to an intravenous ACT yet at the expense of a more invasive approach. In animal models of subarachnoid hemorrhage, intracisternal or intraventricular injection of nimodipine-containing microparticles resulted in more cerebrospinal fluid (CSF) bioavailability compared to intravenous administration (Fowler et al., 2020). Similar to intrathecal baclofen pump to alleviate autonomic disorders and spasticity in severe TBI (François et al., 2001), the development of alike pumps of immune cells, such as Tregs or regulatory B cells, sounds theoretically promising in acute TBI but requires further preclinical and clinical investigation. Several studies reported the beneficial effects of intra-CNS injections of various types of stem cells after TBI (Bonilla and Zurita, 2021). A similar approach using immune cells is currently lacking and the direct immunomodulation via intra-CNS injections is a field that requires further research.

Among the advantages of direct intra-CNS injections is that it allows for precise delivery of a substance with the ability to control the dose and timing of injection. Invasiveness, ethical concerns, and technical expertise are among the challenges of CNS injections.

## Cell depletion therapy

Cell depletion therapy (CDT) has garnered much attention after the success of preclinical and clinical B-cell CDT trials in autoimmune diseases such as multiple sclerosis (Hauser et al., 2008), pemphigus vulgaris (Joly et al., 2007), and rheumatoid arthritis (Edwards et al., 2004). While the pathogenesis of autoimmune diseases differs from that of TBI, the generation of autoantibodies after TBI is a known phenomenon (Zhang et al., 2014; Nasser et al., 2016). Classically, following the activation of T cells as described above, B cell activation is believed to occur via a cognate B cell-T cell interaction that take place in the germinal centers of secondary lymphoid organs (Lee et al., 2021). Those T cells express key costimulatory proteins, such as CD40L, and secrete cytokines that guide the B cell response (Lee et al., 2021). Some of the reported autoantibodies after TBI include those against antigens such as glial fibrillary acidic protein (Zhang et al., 2014) and S100B (Zhang et al., 2014).

Cell depletion therapy (CDT) may be achieved using pharmacological agents that may be administered intravenously or intraperitoneally. For example, an anti-CD19 antibody may be used to deplete B cells (Zhang et al., 2023) and anti-Gr-1 antibody may be used to deplete neutrophils (Stirling et al., 2009). The development of transgenic animals has allowed for the contribution of particular cell types to be studied, thereby providing confirmatory evidence to the pharmacological experiments. For example, microglia depletion can be achieved using several approaches such as clodronate-containing liposomes as well as using *Cx3cr1^Cre^* transgenic mice (Basilico et al., 2022). However, genetic cell deficiency since birth has several limitations particularly pertaining to the maturation and differentiation of those cells. To solve this problem, transgenic mice using an inducible depletion of cells, using the diphtheria toxin receptor (DTR) or the estrogen receptor, can be used. In some animal models of TBI that do not involve direct physical disruption of the BBB (such as the weight-drop model), such experiments may require one to perform intrathecal, intraventricular and/or intraparenchymal injections of depleting agents to ensure adequate therapeutic concentrations are achieved. Experimental findings observed secondary to pharmacological CDT may ultimately be confirmed using animal models that are genetically deficient in particular cell population(s).

A decent number of studies employed the cell depletion approach to investigate TBI neuroimmunology. These studies, as described below, include the depletion of CD8 T cells, macrophages/microglia, γδ T cell subsets, Tregs, CD25+ T cells, neutrophils, Cd11b+ cells, and macrophages/monocytes (Table 1).

Regarding B cells, their depletion was found to reduce neuroinflammation in spinal cord injury (Casili et al., 2016); the data in animal models of stroke has been inconsistent (Malone et al., 2023). For example, B-cell deletion was initially shown to exacerbate pathology and functional deficits acutely after stroke (Ren et al., 2011), whereas later studies revealed that the pharmacological CDT of B-cells did not affect acute infarct volumes/functional outcomes (Schuhmann et al., 2017). In TBI, B-cell CDT, as an interventional therapeutic trial (for example, using depleting antibodies), has not been explored yet. However, direct intraparenchymal administration of exogenous mature and naïve B cells, as detailed in the direct CNS-injections section above, was associated with structural and functional neuroprotection after TBI (Sîrbulescu et al., 2019). In fact, mice genetically devoid of B cells demonstrated a heightened neuroinflammatory profile after TBI (Daglas et al., 2019). It is therefore hypothesized that, based on B-cell deficient models and direct CNS B-cell ACT methods, B cells may be neuroprotective and their depletion may worsen neuroinflammation and neurodegeneration. However, further research is needed to dichotomize the roles of specific subsets of B cells, such as regulatory B cells, in a tissue- and time-specific fashion, and across the various phases of TBI.

Contrary to B-cell depletion, reduced lesion volume(s) were reported after selective depletion of CD4 or CD8 T cells as well as with combined total T + B cell deficiency in animal models of stroke (Hurn et al., 2007; Liesz et al., 2011). In TBI, it was found that depletion of CD8^+^ T cells but not CD4^+^ T cells improved neurodegeneration and neurological outcomes (Daglas et al., 2019). However, given that a protracted increase in CD8^+^ T cells in the injured brain was preceded by infiltration of IL-17-producing CD4 T cells, it is believed that the CD4 Th17 response ultimately drives cytotoxic T cell activity after TBI (Kebir et al., 2007; Braun et al., 2017). In terms of γδ T cells, our group showed that the depletion of Vγ4 γδ T cells ameliorated neuroinflammation secondary to TBI whereas depletion of Vγ1 γδ T cells worsened neuroinflammation (Abou-El-Hassan et al., 2023). Regarding Tregs, diphtheria toxin-induced depletion of Tregs increased CNS T cell infiltration, reactive astrogliosis, IFN-γ signaling as well as improved functional deficits after TBI (Krämer et al., 2019). Depleting Tregs using an anti-CD25 antibody after TBI led to a decrease in lesion size and a concurrent improvement in functional deficits via IL-33 after CCI (Xie et al., 2022). Similar detrimental findings related to Treg depletion have been shown in experimental stroke (Liesz et al., 2009). In summary, these CDT preclinical studies provide evidence on the cell-specific roles of adaptive immune responses in TBI.

In addition to lymphocytes, myeloid cells such as neutrophils, monocytes, and macrophages are among the first responders after TBI and as such accumulate at sites of injury within hours post-injury (Soares et al., 1995; Jassam et al., 2017). Moreover, the severity of injury has been shown to correspond to the number of neutrophils recruited to the injured brain parenchyma (Clark et al., 1994). Despite such associations the precise mechanisms played by neutrophils in the pathobiology of TBI have yielded inconclusive results. For example, although an increase in neutrophils has been observed together with BBB breakdown and neurodegeneration, attempts to deplete neutrophils have failed to establish a direct link between neutrophils and the loss of BBB integrity (Whalen et al., 1999). However, neutrophil depletion has in fact been shown to decrease edema, apoptotic cells, and macrophage/microglia activation at 24 and 48 h after brain contusion (Kenne et al., 2012). In line with such findings neutrophil elastase-knockout mice exhibited diminished edema at 24 h after TBI (Semple et al., 2015). Interestingly, depletion of CD11b^+^ cells, including monocytes, macrophages, and microglia, did not affect lesion size and/or axonal damage after TBI in a valganciclovir-inducible model of CD11b^+^ CDT (Bennett and Brody, 2014). Conversely, diphtheria toxin-induced depletion of CD11b^+^ cells in transgenic CD11b-DTR mice resulted in an increase of proinflammatory gene expression in both the ipsilateral and contralateral hemispheres (Frieler et al., 2015); likewise, microglia depletion in an animal model of stroke resulted in an enlarged ischemic lesions (Marino Lee et al., 2021). Depletion of microglia did not affect the extent of injury-induced traumatic axonal injury suggesting that microglia activation may be important for phagocytosis of apoptotic neurons (Hanlon et al., 2019). Furthermore, it was recently discovered that the depletion of highly phagocytic CD45*^hi^*CD11b*^hi^* monocytes/macrophages abrogated the neuroprotective effects of B-cell ACT (Dwyer et al., 2023). Overall, it appears that neutrophils play a detrimental role and their depletion may be neuroprotective whereas depletion of CD11b^+^ cells is detrimental.

Similar to ACT, among the advantages of CDT is that it allows for targeted investigation of a particular cell population of interest and is currently in use in autoimmune and cancer treatments. Off-target effects, immune suppression, complexity, and ethical considerations are among the challenges of CDT.

## Genetic engineering

Other *in vivo* experimental approaches in neuroimmunology involve the use of genetically engineered animal models using the Cre-*loxP* system and CRISPR/Cas9 as well as employing lentiviruses for gene silencing. The Cre-*loxP* system is the most commonly used breeding system to generate genetically engineered animals (Nagy, 2000). Mouse models are preferred due to their rapid breeding as well as their resemblance to genetic and pathophysiological mechanisms in humans (Perlman, 2016). The Cre-*loxP* system allows researchers to investigate genes of interest in controlled conditions via cell/tissue-specific (spatial control) and/or time-specific (temporal control) manner. The system is often used to make knockout alleles, but it can also be used to activate gene expression.

Mammalian gene editing using the Cre-*loxP* system is universally used and represents a powerful technology for the generation of mutant strains that are in use today in almost all fields of biomedical research. Briefly, Cre (Cre recombinase) is one of the tyrosine site-specific recombinases that recognizes specific DNA fragment sequences referred to as *loxP* (locus of x-over, P1) sites and mediates site-specific deletion of DNA sequences between two *loxP* sites (the floxed DNA segment) (Sauer and Henderson, 1988). Once a Cre recombinase recognizes two directly repeated *loxP* sites, the Cre excises the *loxP*-flanked (floxed) DNA segment. To generate a cell/tissue-specific mutant model, Cre recombinase is designed to be expressed by a promoter that specifically targets the cell or tissue of interest. For example, some of the commercially available promoter-driven models that may be used in studying the neuroimmunology of TBI include Aldh1l1-Cre, CD11b-Cre, CD4-Cre and TCRδ-Cre. Breeding the Cre-driver strain with a floxed mouse strain results in the desired conditional knockout mutant. Tissue specificity is determined by the used promoter and temporal specificity can be achieved using an inducible Cre-*loxP* system. Induction via the exogenous administration of tamoxifen (Brocard et al., 1997) or tetracycline (Gossen and Bujard, 1992) is often employed to allow time-specific gene knockout. This is a critical advantage from a neuroimmunology standpoint since global gene knockout since birth may affect the proliferation and differentiation of immune cells. It is important to mention that appropriate controls must be used to account for the effect of the inducer itself.

A summary of the used Cre promoters of cells of the nervous and immune systems has been summarized elsewhere (Kim et al., 2018). Notably, specific gene knockout in astrocytes may be achieved using the *Aldh1l1^Cre^* strain (Winchenbach et al., 2016) and in microglia using the *Cx3cr1^Cre^* strain (Goldmann et al., 2013, 2016). For example, to study the cell-specific effect of *Apoe* in TBI, genetic deletion of *Apoe* in microglia or astrocytes can be achieved by crossing *Apoe^fl/fl^* animals with *Cx3cr1^Cre^* or *Aldh1l1^Cre^*, respectively. On the other hand, specific gene knockout in αβ CD4 T lymphocytes may be achieved using the *CD4^Cre^* (Lee et al., 2001) and in γδ T cells using the *TCR*δ*^Cre^* strain (Zhang et al., 2015). Similarly, to study the cell-specific effect of *Cd40l* in TBI, genetic deletion of *Cd40l* in αβ CD4 T cells can be achieved by crossing a *Cd40l^fl/fl^* strain with a *CD4^Cre^* strain.

Few studies used the Cre-*loxP* system in experimental TBI (Table 1). Global tamoxifen-induced deletion of HMGB1 after TBI resulted in a significant reduction in the volume of the TBI contusion (Aneja et al., 2019). Furthermore, selective deletion of the Na^+^/H^+^ exchanger (NHE1) gene in microglia using a tamoxifen-induced Cx3cr1^*CreERT*2^:Nhe1*^fl/fl^* strain resulted in accelerated oligodendrocytes regeneration as well as accelerated functional recovery (Song et al., 2022). It is noteworthy to mention that in some instances, deletion of a specific gene in different cell types may result in opposing effects. For example, the silencing of IFN-γ signaling using an astrocyte-specific lentivirus resulted in attenuated neuroinflammation whereas silencing of IFN-γ signaling using a microglia/macrophage-specific lentivirus worsened disease severity in an animal model of multiple sclerosis (Ding et al., 2015). It is therefore crucial to comparatively investigate whether the deletion of genes of immunological significance expressed by neuroglia, such as *Hmgb1* and *Nhe1* among other, would influence chronic neurodegeneration and long-term neurocognitive deficits after TBI.

Other genetic engineering approaches include the use of CRISPR/Cas9 technology (Cota-Coronado et al., 2019). We found one study that used CRISPR/Cas9 gene editing in TBI (Swanson et al., 2020); however, no studies exist that employed tissue- (Meltzer et al., 2019) or cell-specific (Hoffmann et al., 2019) CRISPR/Cas9 in TBI neuroimmunology.

A burdening therapeutic modality in the TBI space relates to the use of short hairpin RNA (shRNA) lentiviruses for gene silencing. Given the role of aquaporin-4 in mediating brain edema after TBI (Xiong et al., 2021), it is perhaps unsurprising that aquaporin-4 shRNA treatment alleviated TBI-induced brain edema, neuronal apoptosis, astrocyte activation and neurological deficits in experimental TBI (Li et al., 2022). Similarly, small interfering RNA (siRNA) targeting AQP4 (siAQP4) resulted in reduced cerebral edema, astrocyte activation and improved motor function (Fukuda et al., 2013). In contrast, delivering the nerve growth factor (β-Ngf) fusion gene using a pseudo lentivirus via a hippocampal injection accelerated cognitive recovery after TBI (Lin et al., 2015). shRNA lentiviruses may be further edited to silence a specific gene on a specific cell type. For example, we designed a GFP-shRNA lentivirus that targets IFN-γR1 specifically on CD11b^+^ cells (Abou-El-Hassan et al., 2023). Via intraventricular injection of this GFP-shRNA lentivirus after TBI, we were able to demonstrate that silencing IFN-γR1 on CD11b^+^ microglia resulted in a more homeostatic microglial profile. Developing nanoparticle platform to improve siRNA permeability across the BBB is a promising tool that may modulate immune responses (Li et al., 2021). Similar lentiviruses may be designed to target specific neuroglia genes of immunological significance in neurotrauma.

Among the advantages of genetic engineering is that it allows for better understanding of the genetic basis of disease as well as precise genetic modifications that have enabled the development of customized pharmaceuticals. Technical expertise, unintended consequences, social acceptance, and regulatory challenges are among the disadvantages of genetic engineering.

## Molecular imaging

Molecular imaging of neuroglia has matured exponentially over the past decades (Kreisl et al., 2020). The development of new radiopharmaceuticals enabled investigations pertaining to the metabolic state of specific neuroglia in health and disease (Crişan et al., 2022). With growing evidence on the pathologic roles of cells of the innate and adaptive immune systems in mediating glial activation and neuronal damage (Daglas et al., 2019; Abou-El-Hassan et al., 2023), employing suitable imaging tools to investigate glial metabolic states can be used as a marker of neurodegeneration after TBI and as tools to measure treatment efficacy (Politis et al., 2012). One of the growing imaging modalities is positron emission tomography (PET) that involves the use of radioactive tracers (Vaquero and Kinahan, 2015). Briefly, radiotracers release positrons that undergo radioactive decay and interact with electrons, resulting in the creation of two photons (Shukla and Kumar, 2006). The PET scanner captures these photons, identified as gamma rays, to generate spatial density images depicting the inner workings and metabolic activities of the brain (Shukla and Kumar, 2006). Customized radioactive tracers, designed for specific neurometabolic processes, enable the detection of these processes (Schwaiger and Wester, 2011). As a result, PET scans employing various tracers offer a non-invasive means of assessing brain metabolism, potentially providing valuable insights into the nature of brain injuries (Bouter et al., 2018).

Translocator protein-18 kDa (TSPO) is one of the molecular biomarkers of microglial activation that have been most commonly used in PET imaging using ^11^C-PK11195 as its radioligand (Debruyne et al., 2003). However, it was found that astrocytes also upregulate TSPO in neuroinflammation limiting its use as a molecular biomarker specific for microglial activation (Chechneva and Deng, 2016). While none is cell-specific, other molecular targets include cyclooxygenase [using ^11^C-Celecoxib (Majo et al., 2005) or ^11^C-Rofecoxib (de Vries et al., 2008)], cannabinoid receptors [using ^11^C-NE40 (Ahmad et al., 2013)], purinergic ion channel receptors [using ^11^C-JNJ-54173717 (Van Weehaeghe et al., 2019)], β-glucuronidase [using ^18^F-FEAnGA (Antunes et al., 2012)], adenosine receptor 2A [using ^11^C-TMSX (Mishina et al., 2011)] and nicotinic acetylcholine receptors [using 2-^18^F-fluoro-A85380 (Martín et al., 2015)] among others. Likewise, tracers of lymphocytes have been driven from engineering fragments of lymphocyte depleting antibodies (for example, YTS169 for CD8 T cells) with radionuclide such as ^64^Cu to generate ^64^Cu-NOTA-YTS169 (Tavaré et al., 2014). As such, ^89^Zr-malDFO-GK1.5 cDb has been used to detect CD4 T cells (Tavaré et al., 2015). While these lymphocyte tracers have been validated in lymphoid organs, their applicability in the CNS compartment is largely unknown (Wei et al., 2018).

Positron emission tomography (PET) molecular neuroimaging has been used across several neurological diseases including stroke (Thiel et al., 2010), Alzheimer’s disease (Passamonti et al., 2018), Parkinson’s disease (Bartels et al., 2010), and amyotrophic lateral sclerosis (Turner et al., 2004) as well as TBI (Folkersma et al., 2011). In a cohort of 18 patients with ischemic stroke who underwent ^11^C-PK11195 PET imaging, persisting tracer uptake in the infarct was found to negatively correlate with clinical outcome (Thiel et al., 2010). Similarly, a higher TSPO signal was detected among 14 National Football League players as compared to controls (Coughlin et al., 2017), that may explain the possible increased risk of development of chronic traumatic encephalopathy among athletes (Jordan, 2013). Furthermore, in a cohort of eight patients with TBI, PET imaging at 6 months revealed widespread increase in ^11^C-PK11195 binding suggestive of diffuse neuronal damage (Folkersma et al., 2011). At the therapeutic level, PET imaging with ^11^C-PBR28 showed that minocycline treatment reduced chronic microglial activation in patients with moderate-to-severe TBI (Scott et al., 2018). Several tracers were developed to detect tau [such as ^18^F-Flortaucipir (Mantyh et al., 2020)] as well as amyloid-beta [such as ^18^F-florbetapir (Wang et al., 2017)]. PET imaging in clinical TBI has been reviewed elsewhere (Huang et al., 2022). Therefore, combining molecular neuroimaging with interventional neuro-immunotherapies (such as ACT or CDT) would help investigate the metabolic recovery of neuroglia after TBI.

Two-photon *in vivo* imaging is another emerging molecular neuroimaging modality that is yet to be employed in TBI neuroimmunology. Two-photon microscopy involves a laser scanning microscopy where a laser beam is directed to a specific location to stimulate fluorescent molecules (Helmchen, 2009). Typically, laser-scanning microscopes capture fluorescence light from that location in the sample at a given instant (Helmchen, 2009). Spatial details are obtained by relocating or “scanning” the laser focus within the tissue. Thus, two-photon *in vivo* imaging requires fluorescence labeling of the structures of interest (Benninger and Piston, 2013). For example, Sword et al. (2013) used FVB/N-Tg(GFAP-EGFP)GFEA-FKi (green-fluorescent astrocytes) and B6.Cg-Tg(Thy1-YFPH)2Jrs/J (yellow-fluorescent neurons) animal strains to demonstrate neuronal and astroglial disruption after experimental TBI along with the decline of peri-contusional cerebral blood flow, while others have demonstrated the use of adeno-associated virus expressing enhanced green fluorescent protein (EGFP) under synapsin promoter to visualize neurons (Zhong et al., 2023). Interestingly, two-photon *in vivo* imaging revealed, besides vasospasm, the formation and clearance of transient microthrombi in capillaries within 1 h post-TBI using an animal model expressing yellow-fluorescent neurons (Han et al., 2020). Likewise, the concept of using transgenic mice expressing fluorescent proteins or the use of viral vectors can be extrapolated toward investigating the role of the adaptive immune response. For example, it may be possible to combine T-cell ACT using CD45.2 cells into a Thy1-YFPH transgenic model to investigate the crosstalk between T cells and neurons with the ability to perform transcriptomic profiling of CD45.2 cells in a CD45.1 host. In either case, the limited availability of cell-specific molecular modalities and multimodal neuroimaging poses a challenge to utilizing advanced molecular tools in TBI neuroimmunology.

Among the advantages of molecular imaging is the nature of its non-invasive approach, its use for early detection of disease and its possible use as biomarker of disease activity and response to treatment. Cost, radiation and contrast exposure, limited availability and complexity of data analysis are among the disadvantages of molecular imaging.

## Co-culture systems and organoids

Unlike the experimental *in vivo* immunology methods described above, several *in vitro* co-culture system models have been devised to study natural or synthetic interactions between cell populations. At the very basic, two or more cell populations are cultured together with some degree of contact. For example, cells may be co-cultured together or physically separated via a membrane such as a transwell co-culture system (Goers et al., 2014). Cell co-culture requires the isolation of the cell populations first. In TBI, microglia are often isolated using a Percoll gradient (Hammond T. R. et al., 2019; Abou-El-Hassan et al., 2023) whereas the isolation of astrocytes may be achieved using a papain-based chemical digestion technique (Wheeler et al., 2020). Using cell-surface staining antibodies and single-cell sorting technology, we recommend the isolation of microglia using the microglia-specific anti-4D4 antibody described by our lab (Butovsky et al., 2012; Krasemann et al., 2017) gated on as live CD45^+^CD11b^+^4D4^+^Ly6C^–^ (Abou-El-Hassan et al., 2023). Astrocytes may be isolated using the ACSA2 cell surface marker (Kantzer et al., 2017). Alternatively, mouse astrocytes expressing green fluorescent protein under the control of the *Aldh1l1* promoter may be used (Sanmarco et al., 2021). For cells of the adaptive immune system, αβ or γδ T cells can be isolated from peripheral immune organs, such as the spleen and deep cervical lymph nodes, using the corresponding cell surface marker. Once the desired cell populations are isolated, cells may be co-cultured per the co-culture system used. Subsequently, cell-culture manipulations may include the addition of exogenous factors or cytokine neutralizing antibodies to investigate a specific hypothesis. RNA-seq is one of the emerging tools in cell biology that enables unbiased transcriptomic profiling of cells. At the conclusion of a co-culture experiment, RNA-seq can be used to detail the molecular profile of the cultured cell populations. Single-cell RNA-seq provides the added benefit of identifying and characterizing the behavior of cell subsets, including novel subsets, within each cell population (Saliba et al., 2014).

Some models include biomimetic environments to re-create, with limitations, the natural milieu including the non-cellular components to aid in resembling an artificial tissue (Goers et al., 2014). For example, organoids are stem cell-derived three-dimensional culture systems that are increasingly used to recapitulate the architecture and physiology of human organs (Kim et al., 2020). Using such organoids, cell migration assays such as the migration of monocytes (Rothhammer et al., 2018) across an artificial BBB allows the investigation of the role of the innate immune system in TBI. Such organoids, such as brain chips, have not yet been employed in experimental TBI neuroimmunology yet. Advanced tissue engineering strategies have enabled the development of pathomimetic models of penetrating TBI composed of astrocytes, microglia, and oligodendrocytes (Liaudanskaya et al., 2020; Basit et al., 2021, 2023) and some included a BBB-like platform composed of endothelial cells and pericytes (Wei et al., 2023). Thus, the use of co-culture systems is useful in certain circumstances such as studying specific cell interactions among cell populations and for further confirming an *in vivo* finding using animal or human cells. Furthermore, since research on human subjects has some limitations particularly in TBI, the use of human cells *in vitro* enables, to some extent, human neuroimmunology investigations. Intercellular communication, paracrine signaling, synaptic signaling as well as chemical and physical cues from the extracellular environment can be assessed among neuroglia and lymphocytes using a co-culture system or an organoid.

Few studies have employed co-culture methods in TBI neuroimmunology (Table 1). Exposing human induced pluripotent stem cell-derived microglia to concentrations of neuroinflammatory cytokines within ranges identified in clinical TBI studies, resulted in a robust microglial response that was found to be more potent compared to that of astrocyte and neuron cultures (Alam et al., 2023). Co-culturing microglia and oligodendrocytes revealed that microglia may modulate the myelinating function of oligodendrocytes in TBI (Wang et al., 2015). Neuroglia cell cultures may also be used for cell death assays, phagocytosis assays (Ritzel et al., 2023) as well as cell viability and tube formation assays (Cai et al., 2022). In addition, we recently confirmed the opposing effects of Vγ1 and Vγ4 γδ T cells on microglia in TBI using an *ex vivo* transwell co-culture system (Abou-El-Hassan et al., 2023). We discovered the upregulation of microglia activation genes in microglia isolated from the TBI group when co-cultured with Vγ1 γδ T cells and anti-TGF-β whereas those co-cultured with Vγ4 γδ T cells and anti-IL-17A neutralizing antibody exhibited downregulation of microglia activation genes. In other transwell co-culture studies, LPS was used to activate microglia and investigate their effect on neuronal apoptosis (Cai et al., 2022). However, isolating microglia directly from TBI brains represents a better recapitulation of TBI pathogenesis as compared to using LPS in cultures. While experimental TBI studies have not used human biofluids *in vitro* to date, co-culture models constitute one of the few methods where human biofluids may be used. For example, culturing neuroglia with CSF isolated from TBI survivors may help investigate the pathways leading to neurodegeneration and identify specific targets for therapy. In fact, it was demonstrated that CSF can maintain the viability and induce proliferation of neural stem cells in *in vitro* cultures (Miyan et al., 2006). Several growth factors, such as fibroblast growth factor and vascular endothelial growth factor, are known to be secreted by the choroid plexus directly into the CSF (Stopa et al., 2001) and these may play a role in cell migration from the ventricular zone to the cortical plate (Ohmiya et al., 2002). Thus, it may be hypothesized that viability promoting factors in the CSF of TBI patients are decreased compared to healthy controls, contributing to the compromised neurogenesis and functional recovery after TBI.

Among the advantages of co-culture systems and organoids is that they allow for cellular crosstalk investigations, disease modeling as well as drug screening. Complexity, cellular heterogeneity, and technical and analytical complexity are among the challenges involving the use of co-culture systems and organoids.

## Current challenges and future directions

Investigating the neuroimmunology of TBI is a daunting task due to the heterogeneity of TBI itself, the dynamic nature of the central and peripheral immune responses and the interplay across neuroglia and immune cells in a time- and location-dependent fashion. This complexity makes it challenging to develop a one-size-fits-all investigative approach to study neuroimmunological aspects all at once. First, understanding the immunological pathogenesis of TBI in experimental models may not necessarily translate into human pathophysiology, limiting the application of promising immunological TBI neurotherapeutics. While animal models are critical for studying TBI, there are challenges in translating findings from animals to humans. Species differences and the inability to fully replicate the complexity of human TBI pose therapeutic challenges. Furthermore, the lack of investigations on some neurovascular compartments poses a challenge for a comprehensive understanding of TBI neuroimmunology. For instance, the leptomeninges, the glymphatics and the BBB are among the biological milieus that often get overlooked and are rarely investigated. The role of meningeal and endothelial cells, as well as mast cells, in acute and chronic TBI is poorly understood and an abundance of knowledge pertaining to alike microenvironments is thought to be lacking. With the development of cell-specific isolation technology, it will be exciting to use recent advances to dissect the role and function of such cells in TBI. From a clinical research standpoint, ethical and practical challenges limit our understanding of the chronic phase of TBI. Research involving TBI patients can raise ethical issues, particularly when considering invasive experimental methods where it can be challenging to recruit and retain participants. In parallel, developing sensitive and specific diagnostic tools for assessing neuroimmunological responses in TBI patients is an ongoing challenge.

Moving forward, employing the substantial advancements in biotechnology and emerging tools would enable detailed investigations at the inter and intra-cellular levels simultaneously. Utilizing some of the neuroimmunology methods detailed in this article, *in vivo* and *in vitro* as well as *ex vivo*, helps provide detailed insights into the neuroimmunological mechanisms after TBI. It will be important to define the unique transcriptional changes that occur in infiltrating and primary CNS cells at different stages of TBI and in different TBI models, in a time and site-specific approach. In this regard, we propose to steer away from the good-bad dichotomy, such as the M1 versus M2 classification for microglia, and rather define the TBI cell-specific inter and intra-cellular networks with a time and site-specific signature. Such approach would guide the development of immunomodulatory therapies to potentiate pathways that dampen neurogenerative programs while promoting tissue remodeling and homeostasis.

## Concluding remarks

**FIGURE 2:**
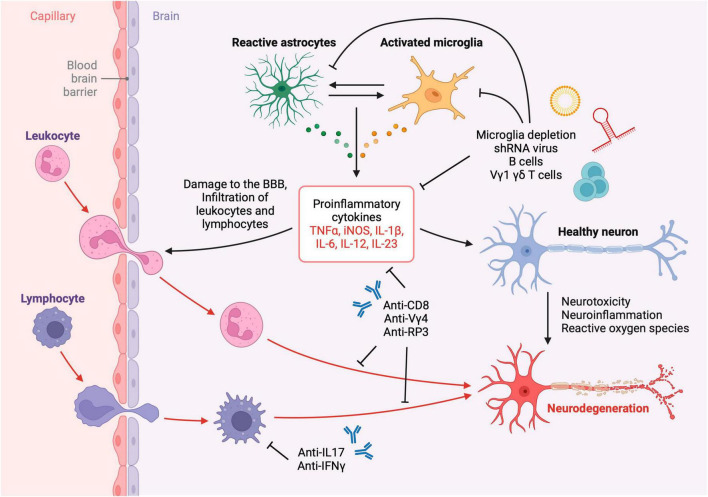
Neuroinflammation and neurotoxicity after TBI. Leukocytes, lymphocytes, and other cells infiltrate the brain parenchyma across the compromised blood-brain barrier after TBI resulting in the activation of resident glial cells and subsequent neurodegeneration. The inflammatory cascade may be attenuated using immunotherapies such as depleting antibodies, neutralizing antibodies, viral therapy and liposomal depletion of glia and immune cells. Created with biorender.com.

Neuroimmunology of TBI is an emerging research field of neurotrauma with a promising translational potential. Accumulating evidence on the roles of the peripheral and central immune responses after TBI have provided new avenues for TBI immunotherapy. These responses are initiated as soon as the initial brain insult occurs and may continue for years leading to chronic neurodegeneration and post-traumatic encephalopathy (Figure 2). Employing methods and approaches to study TBI neuroimmunology, such as the ones highlighted in this article, across the natural course of a brain injury have enabled the discovery of the roles of lymphocyte subsets in TBI. Beneficial aspects of CNS immunity following brain injury take place through innate and adaptive immune mechanisms such as phagocytosis of dead neurons, resolution of inflammation and release of growth factors. Designing strategic experimental investigations using the described neuroimmunology techniques will help further identify the mechanisms of TBI neuroimmunology and guide the development of efficacious TBI immunotherapies.

## Author contributions

HA-E-H: Writing – original draft, Writing – review & editing. JB: Writing – original draft, Writing – review & editing. JC: Writing – original draft, Writing – review & editing. TY: Writing – original draft, Writing – review & editing. RR: Writing – original draft, Writing – review & editing. HW: Supervision, Writing – original draft, Writing – review & editing. SI: Supervision, Writing – original draft, Writing – review & editing.
